# High-performance internal circulation anaerobic granular sludge reactor for cattle slaughterhouse wastewater treatment and simultaneous biogas production

**DOI:** 10.1186/s12896-024-00849-2

**Published:** 2024-05-08

**Authors:** Mohamed Saad Hellal, Hala M. El-Kamah, Hala Salah Doma

**Affiliations:** Water Pollution Research Department, National Research Centre, 33 El Behooth St, Dokki, Cairo, P.O. Box 12622, Egypt

**Keywords:** Anaerobic digestion, Biogas, Slaughterhouse wastewater, Treatment, Methane, Internal circulation, Granular sludge

## Abstract

This research investigates the efficacy of a high-performance pilot-scale Internal Circulation Anaerobic Reactor inoculated with Granular Sludge (ICAGSR) for treating cattle slaughterhouse wastewater while concurrently generating biogas. The primary objective is to assess the efficiency and performance of ICAGSR in terms of organic pollutant removal and biogas production using granular anaerobic sludge. The research methodology entails operating the ICAGSR system under ambient conditions and systematically varying key parameters, including different Hydraulic Retention Times (HRTs) (24, 12, and 8 h) and Organic Loading Rates (OLRs) (3.3, 6.14, and 12.83 kg COD/m³. d). The study focuses on evaluating pollutants’ removal and biogas production rates. Results reveal that the ICAGSR system achieves exceptional removal efficiency for organic pollutants, with Chemical Oxygen Demand (COD) removal exceeding 74%, 67%, and 68% at HRTs of 24, 12, and 8 h, respectively. Furthermore, the system demonstrates stable and sustainable biogas production, maintaining average methane contents of 80%, 76%, and 72% throughout the experimental period. The successful operation of the ICAGSR system underscores its potential as a viable technology for treating cattle slaughterhouse wastewater and generating renewable biogas. In conclusion, this study contributes to wastewater treatment and renewable energy production by providing a comprehensive analysis of the ICAGSR system’s hydrodynamic properties. The research enhances our understanding of the system’s performance optimization under varying conditions, emphasizing the benefits of utilizing ICAGSR reactors with granular sludge as an effective and sustainable approach. Identifying current gaps, future research directions aim to further refine and broaden the application of ICAGSR technology in wastewater treatment and renewable energy initiatives.

## Background

Due to the increasing effects of environmental pollution, there is a growing need for effective treatment methods for industrial wastewater. Particularly in the food production industry, cattle slaughterhouses are major sources of large amounts of wastewater containing organic pollutants and nutrient-rich components [1]. This diverse mixture of waste are derived from various processing and cleaning procedures, such as the accumulation of fat, blood, urine, feces, soil from skin, residual soft tissue fragments from trimming, and cleansing chemicals [2]. Untreated discharge of such wastewater leads to significant public health and environmental hazards, with developing countries being disproportionately impacted [3]. Without proper remediation techniques, this wastewater will contribute to severe environmental contamination, including water body eutrophication and the emission of greenhouse gases [4].

To effectively mitigate these adverse effects, it is essential to implement sustainable and efficient wastewater treatment methods. Common treatment modalities include but are not limited to, chemical treatment, biological treatment, electrochemical treatment, and advanced oxidation processes. Biological treatment approaches involve anaerobic or aerobic processes or a combination them [5]. While aerobic treatment systems are known for their efficiency, they necessitate substantial spatial allocation, maintenance, management, and energy consumption for artificial oxygenation [6]. In this regard, anaerobic digestion appears as a robust and widely acknowledged approach for treating organic-rich wastewater [7, 8]. This biological process involves the microbial breakdown of organic materials in an oxygen free environment, ultimately yielding biogas that comprising methane (CH_4_) and carbon dioxide (CO_2_) [9, 10]. Several types of anaerobic reactors such as up-flow anaerobic sludge blanket (UASB) [11], sequencing batch reactor (SBR) [12], and expanded granular sludge bed (EGSB) reactors [13] have been extensively investigated for the treatment of slaughterhouse wastewater. Even anaerobic digestion can be integrated with electrochemical techniques in microbial fuel cells for bio-waste treatment and methane production [14]. Currently, UASB is considered the most widely applied anaerobic treatment for such wastewater [15]. Nevertheless, the basic design of UASBs proves inadequate for the effective treatment of such wastewater, primarily due to their high organic loading rate (OLR). However, UASB reactors inherently suffer from limitations in mixing and mass transfer within the reactor volume. The lack of efficient mixing can lead to uneven distribution of organic substrates and microbial biomass, affecting the overall anaerobic digestion process. They may also experience challenges in retaining microbial biomass, leading to the washout of valuable microorganisms [16]. These limitations can result in decreased treatment efficiency and the loss of active microbial communities essential for the anaerobic digestion of slaughterhouse wastewater that known for its heterogeneous composition and challenging-to-degrade components. Consequently, the development and optimization of reactors tailored to facilitate efficient slaughterhouse wastewater (SWW) treatment is necessary. A recent modification to the UASB reactor involved the incorporation of internal circulation (IC) of wastewater within the reactor, thereby enhancing the mixing and interaction between the wastewater and the anaerobic biomass [17]. Granular sludge is widely recognized as an effective microbial carrier in IC reactors [18]. It provides a high surface area for biofilm formation and offers advantages such as excellent biomass retention, resistance to shear forces, and tolerance to toxic compounds. The IC mechanism enhances the mixing of wastewater and sludge, promoting efficient substrate utilization and ensuring uniform distribution of nutrients, and this design enables efficient biomass retention, reducing the loss of valuable microorganisms and allowing for higher volumetric loading rates [19]. The hydraulic process within the IC entails the separation of biogas from the liquid midway through the reactor via an integrated gas‒liquid–solid separator (GLSS) device. The separated biogas is subsequently extracted from the system, while the sludge-water mixture descends back to the reactor’s base through an alternate drain. Notably, the increasing forces exerted by the collected biogas recirculate liquid and granular sludge across the lower expanse of the reactor, resulting in an enhanced interaction between the sludge and wastewater.

Although IC anaerobic reactors with granular sludge have been studied extensively for diverse high-strength industrial wastewater, including those originating from paper mills [17], sugar industry [20], distillery and fermentation industry as well as chemical industry [19]. However, there is a lack of studies on the application of such IC reactors with granular sludge for cattle slaughterhouse wastewater (CSWW) treatment. Also, Several studies have investigated the implementation of granular sludge in other anaerobic reactors, such as UASBs [15] and Static Granular Bed Reactors (SGBRs) [21], for poultry SWW treatment.

This study introduces the incorporation of internal circulation (IC) within the reactor, coupled with the use of granular sludge, as a modification to the widely applied UASB reactor. The objective of this study is to provide a comprehensive understanding of the hydrodynamic properties and performance characteristics of pilot-scale internal circulation anaerobic granular sludge reactors (ICAGSR) for the treatment of CSWW and the associated production of biogas. Furthermore, the study investigated the stability, efficiency of biomass retention and resilience of the reactor under various operational conditions, which are crucial factors for the practical use of the reactor on the industrial scale. This aims to address the limitations posed by elevated organic loading rates in traditional systems, showcasing the potential of ICAGSR as an efficient and tailored solution for slaughterhouse wastewater treatment.

## Materials and methods

### Collection and pretreatment of cattle slaughterhouse wastewater (CSWW)

The CSWW was sourced from a discharge point at an abattoir near the Giza governorate in Egypt. The slaughterhouse processes 3–5 metric tons of cattle meat daily, with a peak season output of up to 9 metric tons per day, generating approximately 10–70 m^3^ of wastewater daily. The collected wastewater was manually screened to remove large objects such as hair, skin, and solids larger than 1 mm before sampling. Pretreated CSWW (1 m^3^) was collected biweekly and stored in a 1 m^3^ polypropylene tank equipped with a centrifugal pump for continuous mixing, preventing wastewater fermentation.

### Establishment and operation of internal circulation anaerobic reactor

**Fig. 1:**
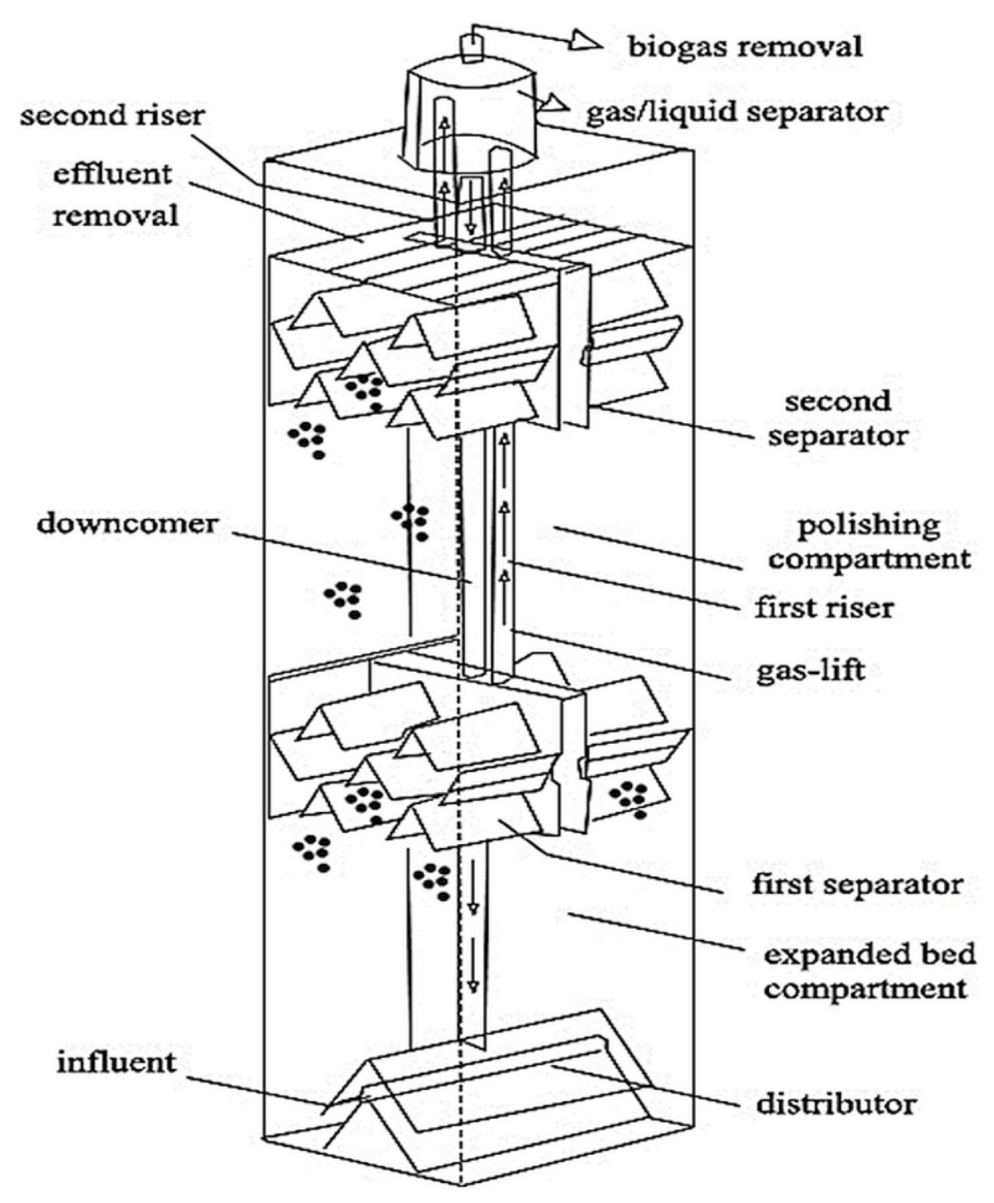
Schematic diagram of the ICAGSR

The internal circulation anaerobic granular sludge reactor (ICAGSR) was constructed from acrylic sheets rectangular in shape and dimensions of 30 cm × 30 cm × 120 cm with a total volume of 100 L. Inside the reactor, as illustrated in Fig. 1, the CSWW was fed through a trigonometry-shaped distributor fixed at the base. Two sets of three-phase separators were placed horizontally at 50 cm and 90 cm from the bottom, accompanied by two sets of riser pipes and one downcomer pipe for fluid movement. The biogas generated within the reactor was collected and separated from water and sludge using three-phase separators, while water and sludge returned to the bottom via internal circulation.

### ICAGSR operating parameters

The ICAGSR was operated in three distinct phases (I, II, and III), each featuring specific flow rates, hydraulic retention times (HRTs), and organic loading rate (OLR) conditions, as summarized in Table (1). A peristaltic pump was utilized for continuous CSWW input.

**Table 1:** Operating conditions and design parameters of the ICAGSR

Parameters	Unit	Phase I	Phase II	Phase III
Flow mode	--	continuous	continuous	continuous
Duration	Days	120	135	120
Flow rate	L/d	100	200	300
HRT	Hours	24	12	8
OLR	kg BOD/m^3^. d kg COD/m^3^. d	1.62 3.3	2.77 6.14	5.87 12.49
HLR	m^3^ /m^2^. d	1.42	2.85	4.3
Temperature range	°C	32–35	30–32	28–33

### Inoculation and start-up

Anaerobic granular sludge was obtained from a full-scale UASB reactor treating maize processing wastewater. The sludge exhibited total solids (TS) and volatile solids (VS) contents of 91 g/L and 82 g/L, respectively. It occupied 25% of the reactor’s total volume. During the start-up phase, a constant influent flow rate of 50 L/d CSWW was maintained for 50 days until steady-state conditions were achieved, as evidenced by consistent chemical oxygen demand (COD) and total suspended solids (TSS) concentrations. The wastewater flow to the reactor then increased incrementally to Phase I flow (100 L/d).

### Specific methanogenic activity of granular sludge

The specific methanogenic activity (SMA) of the granular sludge was examined according to the methodology described by Aquino et al. [22], in which macronutrients (nitrogen, phosphorus, and sulfur) and trace elements necessary for bacterial growth were incorporated. Three 0.5 L sealed serum bottles were used for the experiment and placed in a temperature-controlled water bath shaker at 30 °C. The bottles were filled with 5% NaOH and connected to a gas collecting system. The study lasted 15 days using volatile fatty acid concentrations (acetic and propionic acid) equivalent to 4 gCOD/L supplemented with macro and micronutrients. Sodium bicarbonate (NaHCO_3_) was used to maintain the pH between 7.0 and 7.5, and each bottle contained 5 g VS/L seed sludge. Biogas production was recorded daily through water displacement measurements.

### Anaerobic biodegradability assay of slaughterhouse wastewater

The assay for slaughterhouse wastewater biodegradability followed a standard protocol [23], utilizing two 2 L sealed flasks equipped with magnetic stirrers to gently blend the biomass with wastewater. One flask served as the control and contained distilled water, while the other contained CSWW. Both flasks were inoculated with 5 g VS/L granular sludge supplemented with nutrients, along with the addition of 1 g NaHCO_3_ per liter. Methane gas production monitoring utilized a modified Mariotte flask filled with a 5% NaOH solution to remove CO_2_ from biogas. The COD of the control and CSWW effluent was assessed daily, and the assay was conducted at ambient temperature matching the ICAGSR operation.

### Wastewater sampling and analyses

Wastewater samples were collected from the raw CSWW and the final effluent from the ICAGSR. For each experimental phase, the samples were collected three times per week for a duration of 18 months (6 months for each phase) and were analyzed according to standard methods for the examination of water and wastewater [24]. The analyses were done in duplicates and included the following parameters: pH, total and soluble chemical oxygen demand (COD), biological oxygen demand (BOD), total suspended solids (TSS), total Kjeldahl nitrogen (TKN), ammonia (NH_3_), total phosphate (TP), and oil and grease. In situ measurements of pH and temperature were performed using a portable pH meter. COD and T.P. were measured by a spectrophotometer (HANNA Spectro). Analyses of ammonia and TKN were carried out using a Gerhardt Digestion and Distillation apparatus (Vapodest 20 sn) while TSS and oil were measured by gravimetric analysis.

## Results and discussion

### Characterization of cattle slaughterhouse wastewater

During this study, various physicochemical characteristics of combined CSWW were analyzed. CSWW originates from sources such as manure, urine, blood, lint, fat, carcasses, and undigested food found in the intestines of slaughtered animals and from the cleaning of facility equipment [25]. The key parameters investigated included BOD, COD, TSS, TKN, and oil and phosphorus levels. Table 2 indicates the average physicochemical characteristics of the CSWW during each phase of the study. The high COD concentrations were due to the significant presence of blood in the wastewater outlet pipe. According to Metcalf and Eddy [26], the measured COD and TSS values classify this wastewater as high strength. Fluctuations in COD, BOD, and TSS levels were observed throughout all three phases; these changes can be attributed mainly to market demand-driven variations in product quantities.

Additionally, wastewater analysis indicated the presence of biodegradable organic matter with a BOD to COD ratio ranging from 0.43 to 0.48 (average: 0.45). The soluble fraction ranged between 44% and 56% (average: 53%). An average COD/TKN ratio of 8.2 was calculated, with values ranging between 4.6 and 11.5. Organic nitrogen constituted 41–60% (average: 49%) of the total nitrogen, signifying a mostly protein-based organic matter composition. High oil and grease concentrations were observed to range between 21 and 188 mg/L; this can be attributed to handling procedures involving intestines and stomach contents [27]. The nitrogen and phosphorus concentrations seemed favorable for anaerobic biological treatment, with an average COD: nitrogen: phosphorus ratio of 100:8.7:1.2, compared to the optimal ratio of 100:1.2:0.17 suggested by [28]. Therefore, wastewater characterization revealed an evident excess of nutrients present in the CSWW.

**Table 2:** Characterization of the CSWW during the different study phases

Parameter	Phase I			Phase II			Phase III		
	Min	Max	Average	Min	Max	Average	Min	Max	Average
pH	6.12	8.4	7.4 ± 0.32	7.0	9.3	7.4 ± 0.43	6.9	7.8	7.3 ± 39
COD (mg/L)	1385	6240	3426 ± 480	1111	7711	3075 ± 578	2460	8325	4293 ± 760
BOD (mg/L)	780	2687	1442 ± 236	421	2783	1425 ± 477	1107	4113	2025 ± 580
TSS (mg/L)	146	940	478 ± 178	114	580	306 ± 171	241	3310	838 ± 360
Ammonia (mg/L)	60	297	189 ± 56	78.4	462	243 ± 61	192	535	286 ± 78
TKN (mg/L)	187	568	323 ± 68	193.2	523	323 ± 72	234.6	710	370 ± 84
PO_4_ (mg/L)	8.3	83.1	43 ± 32	6.5	147	69 ± 45	22.4	139	52 ± 23
Oil & grease (mg/L)	21	123.2	58 ± 23	21.2	76.3	49.6 ± 22	38.5	188	77 ± 31

### Specific methanogenic activity of the granular anaerobic sludge

**Fig. 2:**
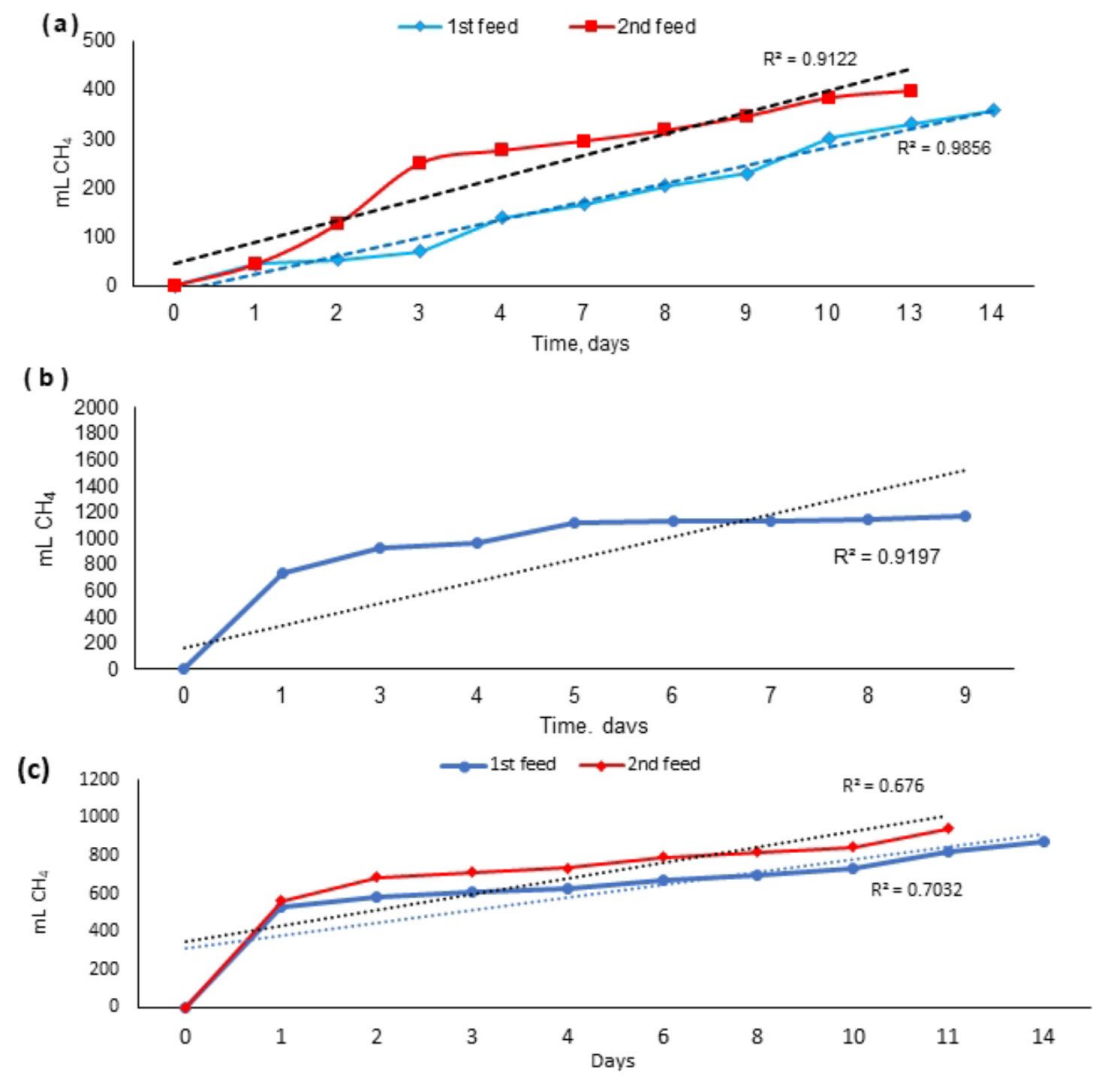
Accumulated gas production during the activity test of granular sludge (**a**) during startup, (**b**) after the 1^st^ phase, and (**c**) after the 3^rd^ phase

**Fig. 3:**
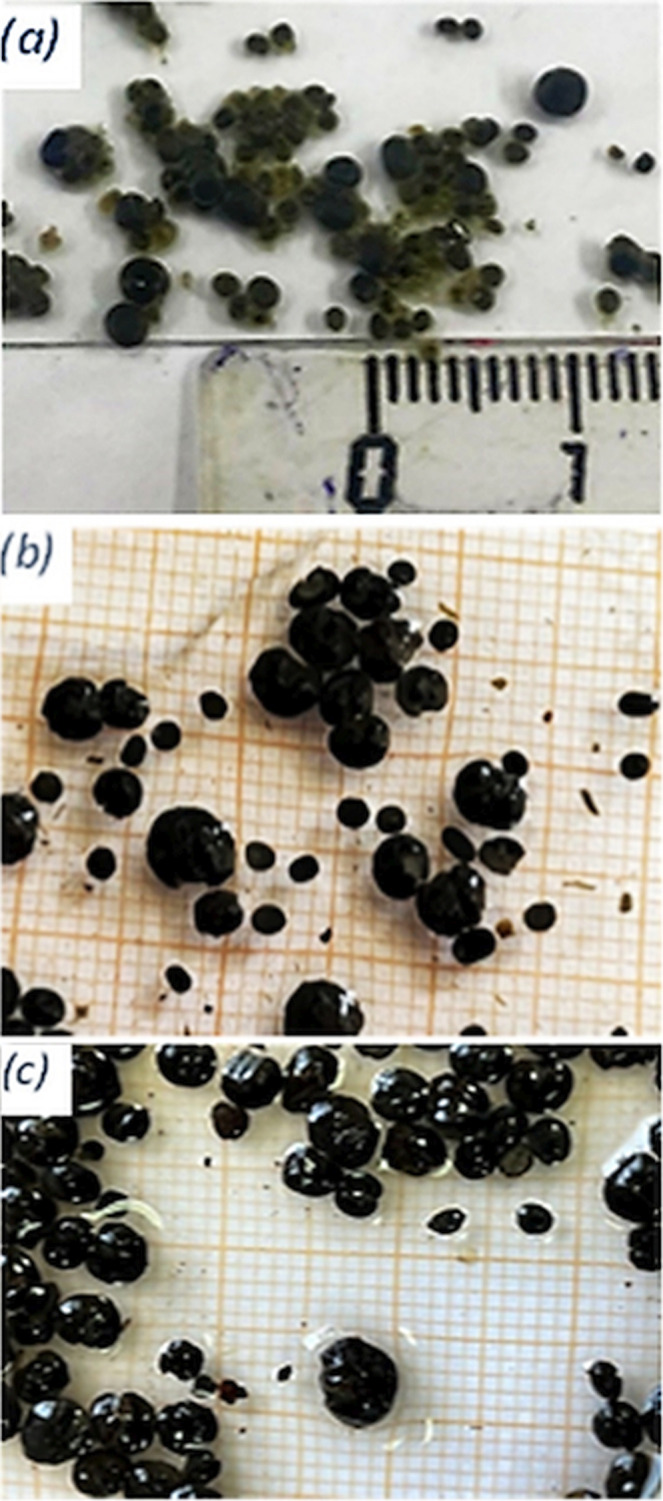
Sludge granule growth before (**a**) start-up, (**b**) after Phase I, and (**c**) after Phase III

The specific methanogenic activity (SMA) is a key for assessing the methane producing ability of sludge for a particular substrate, where substrate availability is not limiting [29]. Determining the SMA during the reactor start-up phase helps to establish the appropriate initial organic loading rate, while monitoring it throughout various stages provides insights into sludge development [30]. In this study, the SMA was measured before reactor start-up, after phase I, and after phase III. The obtained results, as shown in Fig. 2 (a, b & c), revealed that the sludge methanogenic activity was initially 399 mLCH_4_/g VS. Following the first phase, the sludge activity remained relatively stable at 380 mLCH_4_/g VS but decreased to 290 mLCH_4_/g VS after the third phase, indicating a decrease of only 100 mLCH_4_/g VS over a continuous operational period of approximately 18 months. Regarding the sludge’s appearance, its diameter was approximately 1 to 2 mm or less during start-up (Fig. 3a). However, after the second and third phases, the sludge granules became larger and more defined and had a diameter ranging from 1 to 5 mm (Fig. 3b & c).

### Anaerobic biodegradability of CSWW

**Fig. 4:**
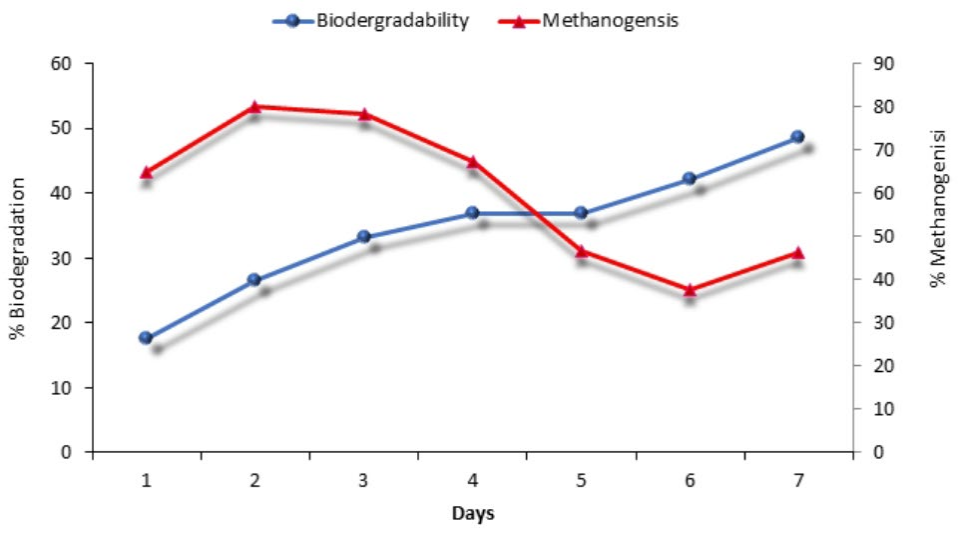
Biodegradability and methanogenesis rate of CSWW

To evaluate the anaerobic biodegradability of CSWW, it was subjected to a 7 days (168 h) experiment at ambient temperature. The data presented in Fig. 4 show that the percentage of biodegradability of CSWW varied between 17% and 48.6%, with an average of 34.5%. The initial methanogenesis rate reached 78.8%, but this value decreased to 37% after 6 days, with an average methane percentage of 62.5%. The lower methanogenic activity observed could be attributed to the high protein concentration in the CSWW, which resulted in the release of inhibitors such as ammonia, fats, and long-chain fatty acids that hindered the methanogenic process.

### Effect of different OLRs and HRTs on the performance of the ICAGSR for CSWW treatments

**Fig. 5:**
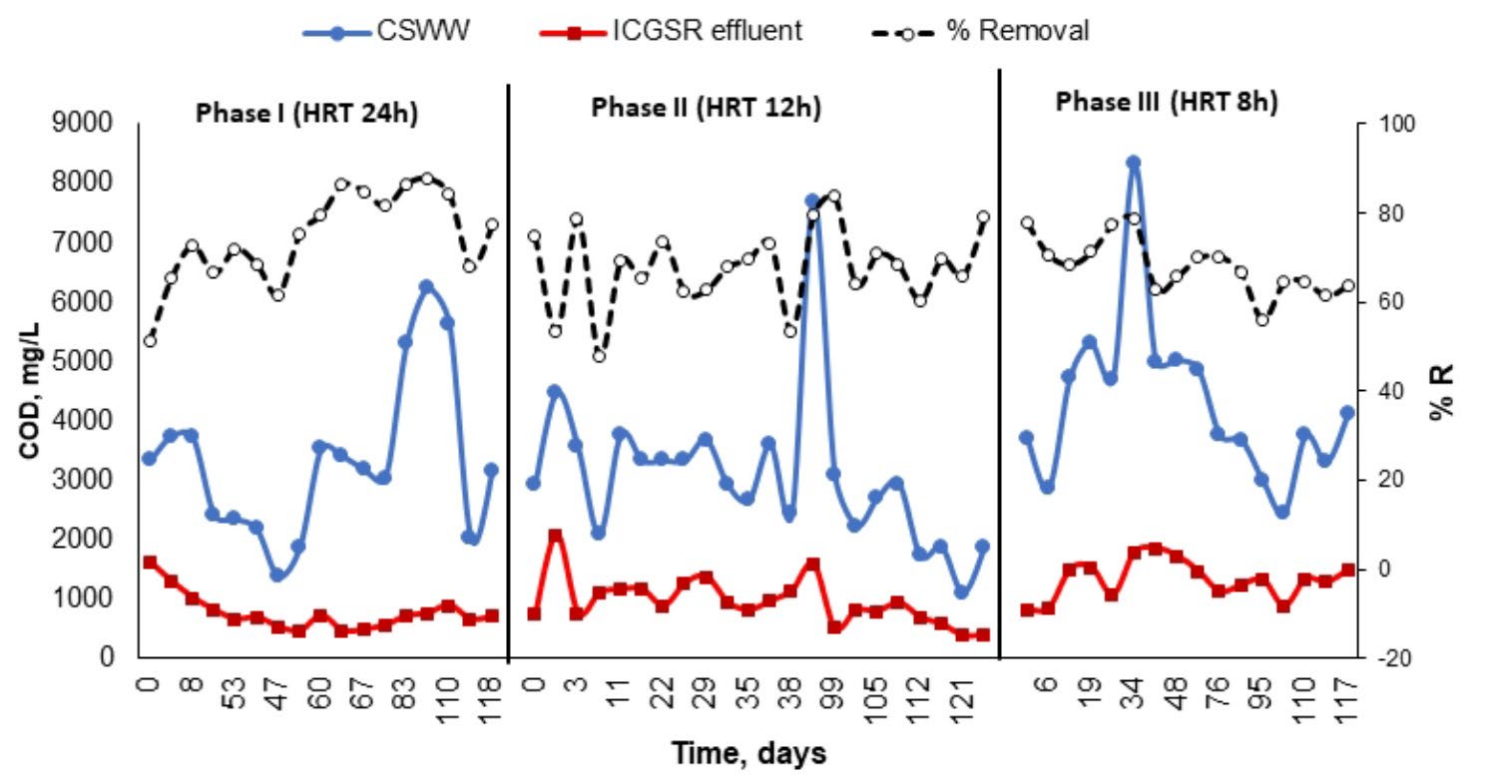
Variation in COD and removal efficiency during the different study phases

**Fig. 6:**
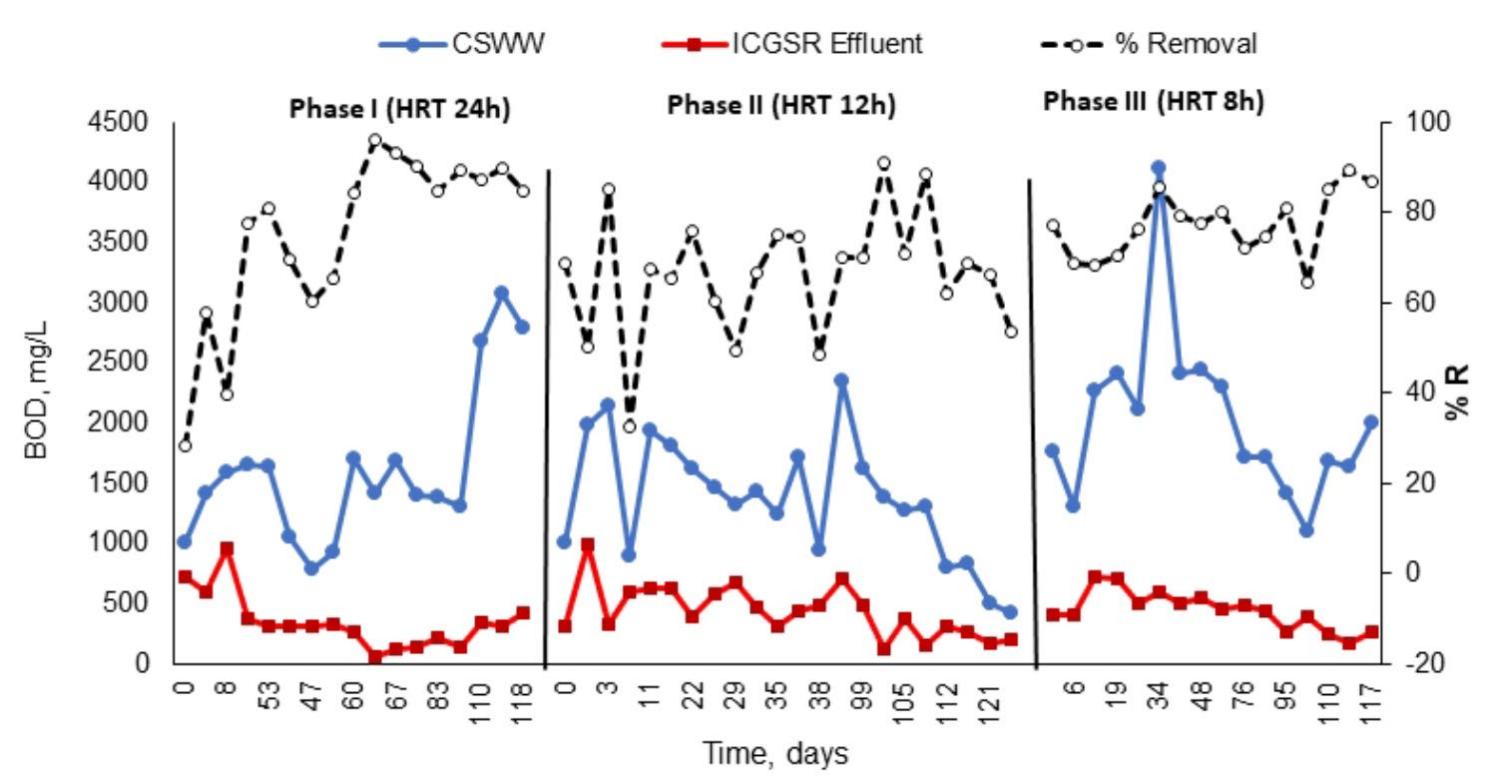
Variation in BOD and removal efficiency during the different study phases

**Fig. 7:**
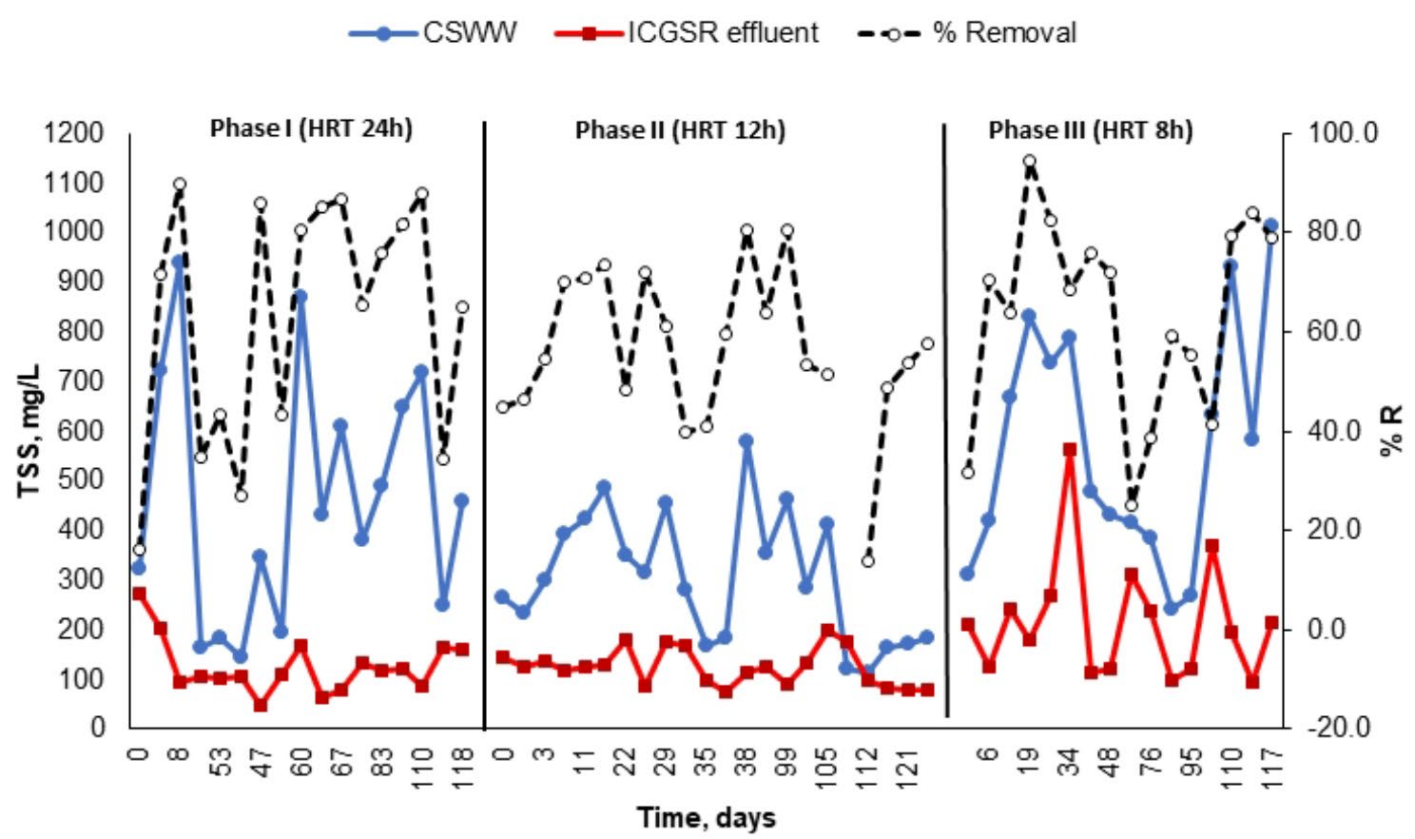
Variation in TSS content and removal efficiency during the different study phases

In the present study, various OLRs and HRTs were assessed within the ICAGSR to ascertain the influence of process parameters (HRT and OLR) on reactor performance and stability. This was done to establish an optimal condition for pollutant removal utilizing a continuous pilot-scale reactor. To achieve this objective, several HRTs (24, 12, and 8 h) and OLRs (3.3, 6.14, and 12.43 kg COD/m^3^. d) were examined. The reactors were initially started at an HRT of 24 h, followed by a decrease to 12 and 8 h. Table 3 shows the average residual concentrations and removal efficiencies across the different phases conducted and the tested HRTs and OLRs for ICAGSR in this investigation, while Figs. 5, 6 and 7 present the variations in specific pollution parameters during each phase.

**Table 3:** Average residual concentration and percent removal of pollutants during different study phases

Parameter	Phase I		Phase II		Phase III	
	Average (mg/L)	% Removal	Average (mg/L)	% Removal	Average (mg/L)	% Removal
pH	7.4	-	7.5	-	7.5	-
COD	766 ± 56	74.7	955 ± 68	71.9	1325 ± 156	68.1
BOD	348.1 ± 33	75.2	437 ± 46	66.3	445 ± 76	72.3
TSS	126 ± 16	63.3	124 ± 19	56.6	216 ± 36	53.9
Ammonia	226.6 ± 38	-	242 ± 29	--	296 ± 39	--
TKN	323.6 ± 42	-	288 ± 37	--	364 ± 46	--
PO_4_	63.0 ± 12.3	69.0	33 ± 6.5	54.2	22.6 ± 3.2	51.5
Oil	18.1 ± 5.2	68.0	21.8 ± 4.3	54.5	35.6 ± 9.1	52.5

As illustrated in Figs. 5, 6 and 7, at an HRT of 24 h, the maximum removal efficiency was with an OLR ranging from 1.4 to 6.24 kg COD/m^3^. d (averaging at 3.3). The mean removal percentages for COD, BOD, and TSS were 74%, 75%, and 63%, respectively, with average residual values of 766 ± 56 mg/L, 348 ± 33 mg/L, and 126 ± 16 mg/L, respectively. The analysis of nitrogen fractions revealed that NH_4_-N concentrations either increased or remained unchanged in the effluent when compared to influent values, reaching up to a 3% increase during this period. Ammonia is produced as a by-product of the biodegradation process, principally from Ammonia is produced as a by-product of the biodegradation process, principally from the mineralization of nitrogen during deamination of protein, and mainly exists in the form of ammonium (NH4^+^) and free ammonia (NH_3_).

When the HRT was reduced to 12 h, the OLR increased to an average of 6.14 kg COD/m^3^. d while ranging between 2.2 and 15.4 kg COD/m^3^.d due to high fluctuations in CSWW COD values (Fig. 5). Despite the increase in OLR, the reactor performance showed only a marginal decline, with the mean COD concentration of the treated effluent ranging from 350 to 2080 mg/L (mean of 954 ± 86 mg/L), and its removal percentage decreased by 7% from phase I (Table 3; Fig. 5).

The average BOD removal percentage decreased by 9% from the initial phase to 66% (Fig. 7), with an average BOD value of 437 ± 46 mg/L in the treated effluent. The TSS concentrations in CSWW were notably high and fluctuated between 117 and 478 mg/L, with a mean value of 124 ± 19 mg/L (Table 3). The average TSS removal percentage varied from 14 to 88%, with a mean of 56% (Fig. 7). The large fluctuations in TSS removal can be attributed to internal circulation, which causes high up-flow velocities within the reactor and consequently leads to disorder and elevated suspended solids concentrations in the ICAGSR effluent.

**Fig. 8:**
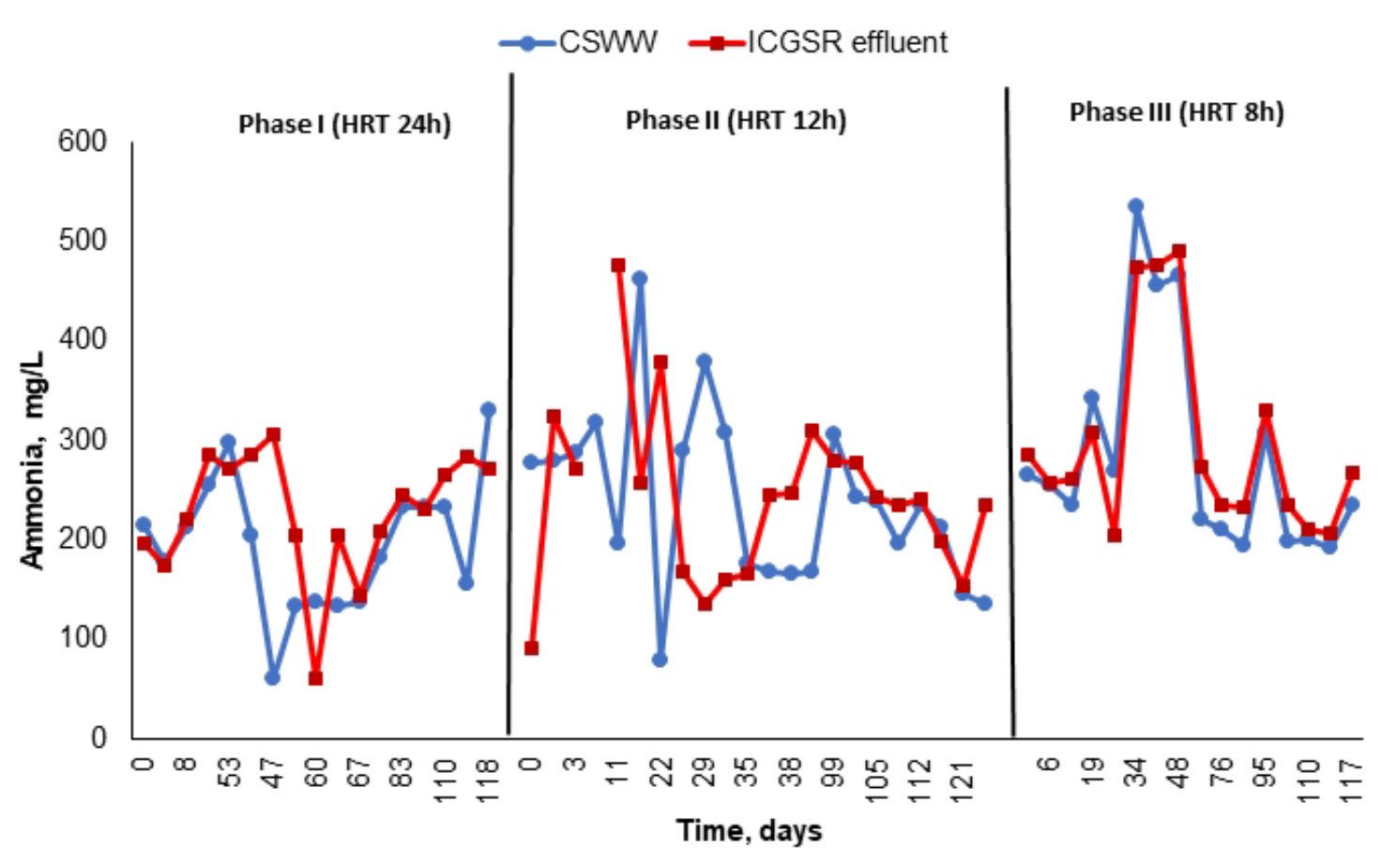
Variation in the ammonia concentration and removal efficiency during the different study phases

In phase II, neither ammonia nor nitrogen compounds exhibited any signs of removal, with some samples even showing ammonia concentrations in the effluent equal to or greater than those in the influent (Fig. 8; Table 3). After a decrease in the HRT to 8 h (Phase III), the organic loading rate increased to an average of 12.85 kg COD/m^3^.day. According to the results in Table 3, the ICAGSR performance remained relatively unaffected by this increase in pollutants load. COD and BOD elimination efficiencies were 68% and 72%, respectively, yielding residual concentration values of 1325 ± 156 and 445 ± 76 mgO_2_ /L, respectively. The average removal percentage for TSS was declined to 53.9%, with an average residual concentration of 216 ± 36 mg/L. The increase of TSS and COD concentrations in the treated effluent indicating that decreasing the HRT may lead to slight washout of insoluble biomass due to the high velocity of wastewater flow. The ammonia concentrations in the effluent exceeded those observed in the previous two phases (Fig. 8). Additionally, the total Kjeldahl nitrogen concentration either maintained or slightly decreased by a mere 2% in the effluent (Table 3). Ammonia nitrogen inhibits anaerobic reactors and usually occurs when concentrations reach 1500 to 3000 mg/L [31].However, the current concentration of ammonia was much below the level of inhibition during the HRT study period.

CSWW contains a high amount of fats, oils and grease, which can cause serious problems such as pipe obstruction and sludge adhesion [2]. In this study, the average removal rate of oil and grease at HRT 24 h was 65% and it slightly decreased to 56% and 52% at 12 h and 8 h. these results are better than those obtained by Musa and Idrus [32] who reported about 55% removal of oil and grease at HRT 24 h while the maximum reduction(80%) was achieved at 48 h.

Numerous prior research studies have demonstrated that both the OLR and HRT significantly influence effluent properties. As a result, COD remains widely recognized as a key parameter for assessing reactor performance and monitoring the effects of these parameters throughout various studies [11, 12, 33, 34]. These studies reported influent COD concentrations (found in raw wastewater) varying between 2000 and 10,000 mg/L for anaerobic treatment processes. The present research aligns with certain findings [15, 35, 36] and surpasses others [37], which required an HRT exceeding 24 h to achieve equivalent efficiency. Despite these similarities, none of the referenced studies incorporated granular sludge within internal circulation anaerobic reactors for treating this specific type of wastewater. To evaluate the potential of ICAGSR for other wastewater sources, a comparative analysis of wastewater chemical composition is essential. Different industrial processes generate unique wastewater profiles, ranging from organic pollutants and suspended solids to specific contaminants characteristic of the respective industries [3]. ICAGSR, with its internal circulation mechanism and use of granular sludge, offers advantages in terms of efficient substrate utilization, biomass retention, and tolerance to toxic compounds. These features make it a potential candidate for the treatment of high-strength industrial wastewater from various sectors such as pulp and paper mills, sugar industries, distilleries, and chemical manufacturing plants. The granular sludge’s resistance to shear forces and its ability to foster biofilm formation contributes to its adaptability to different wastewater compositions [38]. The flexibility of ICAGSR design allows for customization and optimization to cater to specific wastewater characteristics. By adjusting operational parameters and reactor configurations, researchers and practitioners can tailor ICAGSR systems to address the unique challenges posed by different industrial effluents.

### Biogas production from ICAGSR under different OLRs

**Fig. 9:**
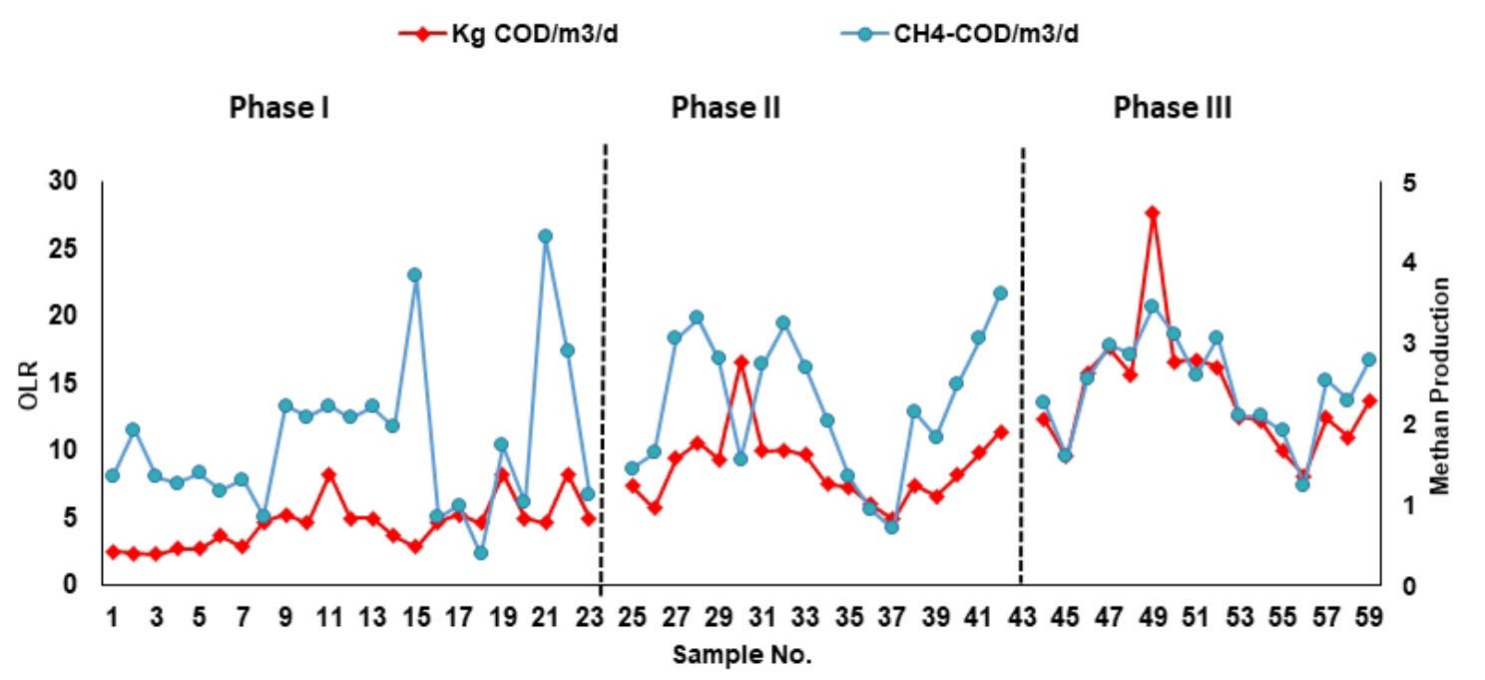
Applied organic load and gas production in the three phases

During phase I at an HRT of 24 h and an average OLR of 3.3 kg COD/m^3^. d, the average methane production was 1.25 kg CH_4_-COD/m^3^. d, and the average volumetric production was 0.5 m^3^/m^3^/day (Fig. 9). The amount of biogas collected from the reactor varied according to the removal of biodegradable matter as well as the organic loading rate applied. Figure 9 shows the quantity of biogas collected daily. The percentage of total influent COD converted into methane ranged between 10% and 60%, with an average of 46%. The percentage of influent COD soluble to methane ranged between 20 and 90%, with an average of 52%. The biogas analysis showed that during this phase, the carbon dioxide concentration ranged between 14 and 16%. The percentage of methane ranged between 78% and 80% (Table 4).

**Fig. 10:**
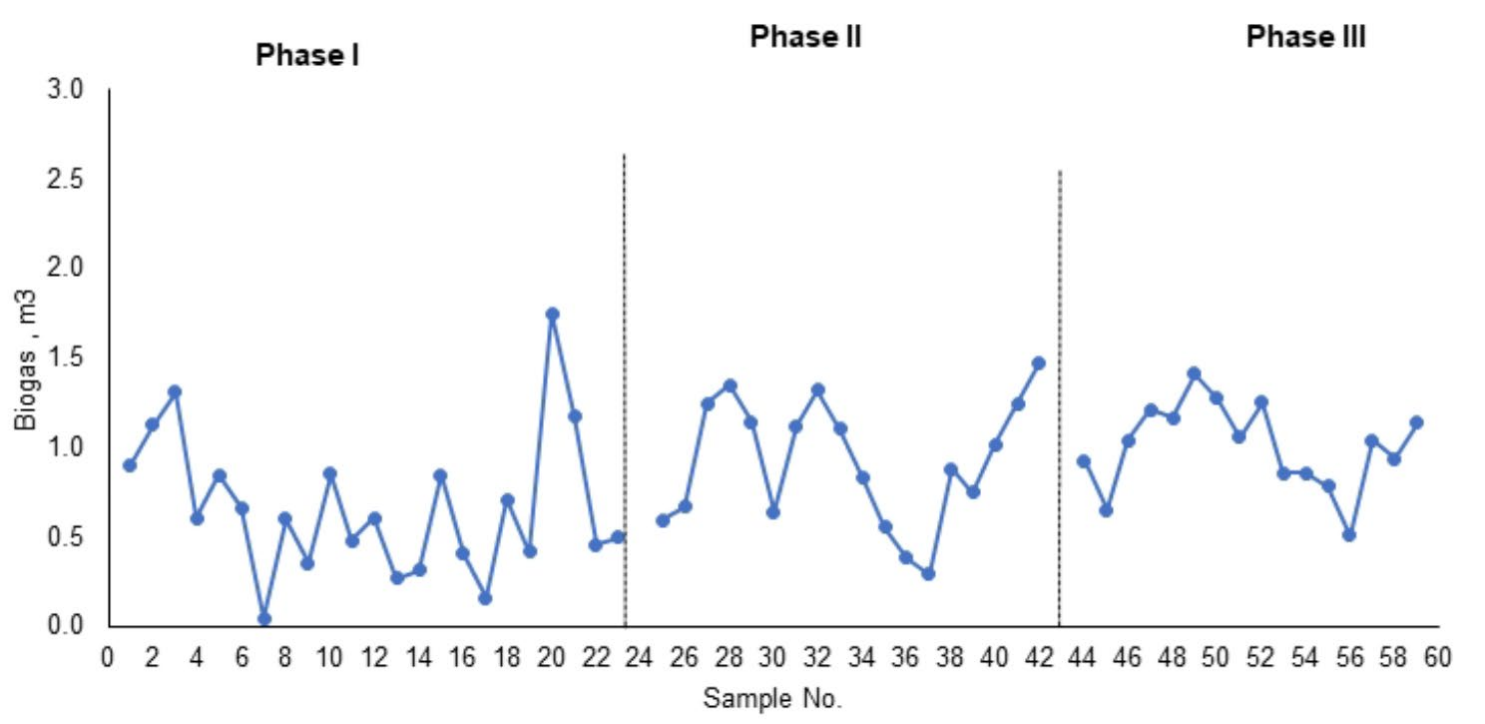
Volumetric gas production m^3^gas/m^3^ wastewater

The organic load increased during phase II to an average of 6.14 kg COD/m^3^. d, and the HRT was 12 h. The gas production rate increased during this phase by approximately 71%. The volumetric methane production rate as a function of the OLR is shown in Fig. 10. The volume of gas produced per day increased with increasing OLR over the range tested, and the volumetric methane produced was equivalent to 0.9 m^3^/m^3^ wastewater. The average methane production rate is 2.3 kg CH_4_-COD/m^3^. d. The activity of methanogenic bacteria was not impaired at higher OLRs. Nevertheless, the analysis of gas content showed that the methane percentage decreased by 68% compared with that in the first load. The CO_2_ and nitrogen percentages increased by approximately 8% and reached an average of 22% and 7.4%, respectively. The percentages of influent total COD and total COD soluble COD converted to biogas were 38 and 51%, respectively. When the COD was removed and converted to methane increased to 77% (Table 4).

In Phase I, with an HRT of 24 h and an average OLR of 3.3 kg COD/m^3^. d, ICAGSR exhibited promising methane production, with an average of 1.25 kg CH_4_-COD/m^3^. d and a volumetric production rate of 0.5 m^3^/m^3^/day (Fig. 9). This finding is consistent with findings from similar studies on anaerobic digestion systems with internal circulation [39]. The daily variation in biogas quantity (Fig. 10) demonstrated a correlation between the removal of biodegradable matter and the applied organic loading rate. However, a nuanced exploration of the biogas composition revealed that the percentage of total influent COD converted into methane ranged from 10 to 60%, with an average of 46%. Similarly, the percentage of influent COD soluble in methane exhibited variability, ranging from 20 to 90%, with an average of 52%. Gas analysis during this phase indicated a carbon dioxide concentration ranging from 14 to 16%, with methane concentrations consistently ranging from 68 to 70% (Table 4). These results align with expectations for anaerobic digestion processes, where methanogenic bacteria play a crucial role in converting organic matter into methane and carbon dioxide [40].

During Phase II, with an increased organic load (average OLR of 6.14 kg COD/m^3^. d) and a reduced HRT of 12 h, the gas production rate increased by 71%. This increase in volumetric methane production (Fig. 10) suggested the resilience of the methanogenic bacteria to higher OLRs. The average methane production rate increased to 2.3 kg CH_4_-COD/m^3^.d, indicating the adaptability of the microbial community to elevated organic loads [41]. However, despite the increased methane production, the gas analysis revealed a decrease in the methane percentage to 56%, a 14% reduction from the first load. This decrease in methane content was accompanied by an 8% increase in carbon dioxide and a notable 7.4% increase in nitrogen. These findings emphasize the importance of monitoring gas composition to understand the microbial response to varying organic loads and potential shifts in metabolic pathways [42]. The percentages of total influent COD and soluble COD converted to biogas were 38% and 51%, respectively, highlighting the dynamic nature of anaerobic digestion under changing operational conditions. In comparison to the findings of other studies, our results underscore the resilience of methane production in the ICAGSR at elevated OLRs. However, the observed decrease in methane percentage during Phase II warrants further investigation into microbial community dynamics and potential shifts in metabolic pathways under increased organic loads, aligning with findings in studies exploring similar anaerobic digestion systems [43].

**Table 4:** The volumetric methane production rate and the percentage of the imposed COD converted into methane

Phase	OLR (kg COD/m^3^. d)	HRT (h)	Methane (kg CH_4_-COD/m^3^. d)	% COD _tot_ to CH_4_	% COD _sol_ to CH_4_	%COD _removed_ to CH_4_
I	4	24	0.7	49%	52%	40%
II	8	12	2.3	26%	76%	70%
III	12.5	8	2.5	18%	41%	77%

When the organic load (OLR) increased to an average of 12 kg COD/m^3^/d during phase III, the large production ratio also increased, and the average biogas production rate reached 2.5 kg CH_4_-COD/m^3^/d. The percentage of total COD supplied to the reactor converted to biogas ranged between 13 and 21%, with an average of 19%. The percentage of soluble COD converted to biogas ranged between 30 and 48%, with an average of 51%. The COD removed during the anaerobic process converted 49 to 78% to methane, with an average of 77%. The analysis of the biogas during this phase showed that the methane percentage decreased to 46%. The decrease in the methane content with increasing OLR might be attributed to the inhibition of methanogenic bacteria at high OLRs. Comparing these findings with those of existing studies provides valuable insights. The observed decrease in methane content aligns with the inhibition hypothesis, which is consistent with the findings of Mel et al. [44], who noted a similar phenomenon under elevated OLR conditions in anaerobic digesters. The results indicated that there was a direct relationship between the OLR and gas production during the three phases, as illustrated in Figs. 9 and 10. Additionally, the positive correlation between the OLR and gas production, as illustrated in Figs. 9 and 10, agrees with the findings of a study conducted by Lins et al. [45], emphasizing the direct influence of the OLR on biogas generation. Compared to other anaerobic reactors used for the treatment of CSWW, the integration of internal circulation with granular anaerobic sludge reactor offers superior mixing and biomass retention, enabling more effective treatment of CSWW with higher organic loads. Table 5 shows a performance evaluation of different anaerobic reactor for CSWW treatment under similar operating conditions. The ICAGSR reactor has the advantage of retaining biomass, which promotes high methanogenesis activity. The system efficiently converted the removed pollutants into methane under high-loading conditions. An increase in the OLR is a matter of considerable concern because long-term loading will be irrevocably accompanied by a high accumulation of slowly biodegradable insoluble substrate ingredients in the sludge, which will decrease methanogenic activity.

**Table 5:** Evaluation of different anaerobic reactors for CSWW treatment

Technology used	Performance	Reference
Modified UASB	68% COD removal and 85% methane content at 24 h HRT and 5 g/L.d OLR	[11]
Anaerobic baffled reactor (ABR)	Maximum COD reduction of 70% and 50–60% methane content at OLR of 0.2 kg COD/m^3^.d and HRT of 43 h	[46]
Anaerobic hybrid reactor (AHR)	79% COD reduction and 65% methane content at OLR of 3 kg COD/m^3^.d and HRT of 24 h	[47]
Anaerobic membrane bioreactor (AnMBR)	62% removal of COD and 64% methane content at 30 h HRT and 13.1 kg COD/m^3^.d	[48]
ICGASR	74% COD removal and 80% methane concentration at 24 h HRT and OLR of 3.3 kg COD/m^3^.d	Present study

## Conclusion

In conclusion, a high-performance pilot-scale internal circulation anaerobic reactor inoculated with granular sludge (ICAGSR) demonstrates significant potential for treating cattle slaughterhouse wastewater and simultaneous biogas production. The study’s findings indicate that the ICAGSR system is capable of effectively removing organic pollutants, achieving removal efficiencies of up to 74% for COD under various hydraulic retention times and organic loading rates. More importantly, the ICAGSR system consistently generates sustainable biogas with a high methane content throughout the experimental period. These results suggest that the utilization of granular anaerobic sludge within an ICAGSR reactor is a promising and sustainable approach for wastewater treatment and renewable energy generation in the cattle slaughterhouse industry. Further research is encouraged to optimize the ICAGSR system’s performance and integration with other treatment techniques for maximum pollutant removal efficiency and biogas production, thereby contributing to advancements in environmental protection and clean energy technologies. While our study demonstrates promising results at the pilot scale, further research should focus on the scalability of the ICAGSR system for industrial applications. Conducting larger-scale trials and pilot projects in collaboration with slaughterhouse facilities will provide valuable insights into the system’s performance under real-world conditions.

## Data Availability

The datasets used and/or analyzed during the current study are available from the corresponding author on reasonable request.
